# Obesity difference on association blood malondialdehyde level and diastolic hypertension in the elderly population: a cross-sectional analysis

**DOI:** 10.1186/s40001-022-00983-7

**Published:** 2023-01-24

**Authors:** Ying Huang, Hong Chen, Qifan Liu, Jinzhu Hu, Dongxi Hu, Zixi Huang, Zhenyan Xu, Rong Wan

**Affiliations:** 1 Rehabilitation department, The Second Affiliated Hospital of Nanchang University, Nanchang, Jiangxi China; 2 Department of Cardiovascular Medicine, The Second Affiliated Hospital of Nanchang University, Nanchang, Jiangxi China; 3 Department of General Medicine, The Second Affiliated Hospital of Nanchang University, Nanchang, 330006 Jiangxi China; 4 Jiangxi Key Laboratory of Molecular Medicine, No. 1 Minde Road, Donghu, Nanchang, 330006 Jiangxi China

**Keywords:** Malondialdehyde, Oxidative damage, Blood pressure, Obesity, ROS

## Abstract

**Aims:**

Although increased production of malondialdehyde (MDA), an end product of lipid oxidation caused by reactive oxygen species (ROS), has been found be elevated in hypertensive population, whether MDA contributed to a changed risk of hypertension is uncertain. We aimed to investigate whether elevated blood levels of MDA contribute to increased risk of hypertension and obesity has a modified effect on the association in an older Chinese population.

**Methods:**

Data were obtained from 2011 to 2012 of the Chinese Longitudinal Healthy Longevity Survey (CLHLS), a national cohort of older adults in China. Associations between blood MDA level and systolic and diastolic blood pressure (BP) and risk of hypertension were performed by multivariable linear regression and logistic regression analysis.

**Results:**

The results of smooth curve revealed a gradual upward trend on association of blood MDA level with diastolic BP (*P* < 0.001), but not with systolic BP (*P* > 0.05). Logistic regression analysis suggested that elevated blood MDA levels were associated with increased risk of diastolic hypertension (*OR* = 1.079, 95% CI 1.039–1.122, *P* < 0.001) rather than systolic hypertension (*OR* = 0.978, 95% CI 0.943–1.015, *P* = 0.247) after adjustments of related confounding factors were made. Furthermore, we found the significant modification effect of obesity on the association between MDA level and risk of diastolic hypertension evaluated by body mass index (BMI, interaction *P* = 0.015) and by waist circumference (interaction *P* = 0.016).

**Conclusion:**

Our results firstly identified that increased blood MDA levels were associated with elevated risk of diastolic hypertension, rather than systolic hypertension in the non-obese old population.

**Supplementary Information:**

The online version contains supplementary material available at 10.1186/s40001-022-00983-7.

## Introduction

Oxidative damage can arise from imbalance between increased production of reactive oxygen species (ROS) and/or reduction of antioxidant defenses [1]. Malondialdehyde (MDA), the main end product of lipid oxidation caused by ROS, can cause cross-linking polymerization of proteins, nucleic acids, and other living macromolecules [2] and do great damage to the activities of mitochondrial respiratory chain complex and key enzymes in mitochondria [3]. At present, a lot of evidences have confirmed that blood MDA is an important indicator of degree of oxidative damage in vivo and in vitro [4].

Some previous studies on the role of oxidative damage in hypertensive patients have been confirmed. In essential hypertension, ROS may act through several mechanisms: (1) a direct action on endothelial cells, resulting in structural and functional damage; (2) degradation of the potent vasodilator nitric oxide (NO); (3) effects on eicosanoid metabolism in endothelial cells; and (4) oxidative modification of low-density lipoproteins [5]. Importantly, obesity and obesity-related diseases, such as hyperinsulinemia, increased mobilization of fatty acids, or increased concentration of catecholamines, frequently found in patients with essential hypertension, have been reported to be involved in the overproduction of ROS [6–8]. High levels of ROS in obese state were intricately linked with higher risk of essential hypertension and other diseases, including type 2 diabetes and ischemic heart disease [9–11]. Mechanism studies have suggested that cellular oxidative stress and subsequent oxidative damage are associated with an oversupply of reducing equivalents in adipose cells [12]. High levels of circulating glucose and lipids can result in an excessive supply of energy substrates to metabolic pathways, which in turn can increase the production of ROS [12]. Ultimately, an imbalance between the production of ROS and the antioxidant defense mechanisms may contribute to the development of essential hypertension.

Although several studies have reported that blood MDA levels were elevated in hypertensive populations [1, 13, 14], whether high blood MDA levels contribute to increased hypertension risk is uncertain. Considering present research background, we determined blood concentrations of MDA in obese and non-obese subjects in a Chinese elderly population from the Chinese Longitudinal Healthy Longevity Survey (CLHLS). Our first aim was to investigate whether blood MDA levels were associated with increased risk of hypertension in an older Chinese population. Then we further investigated whether obesity had a modified effect on the association between blood MDA level and hypertension risk in the present study.

## Materials and methods

### Study population

Study data were obtained from the CLHLS study [15], which is a prospective, longitudinal, community-based study. 22 provinces in China were initially selected and then half of the cities or counties in these provinces were randomly selected in to included study population. A detailed description of the CLHLS has been also published elsewhere [16]. In summary, this national cohort was performed on an old population and the cohort study has high-quality data with demographic and socioeconomic characteristics, social and behavioral risk factors and health indicators such as blood test and urine test. Further details on study design, study procedures and data quality assessment were performed elsewhere [17–19]. All participants or their relatives were informed of the systolic and diastolic blood pressure (BP) results and use of the data for research in CLHLS study. Written informed consent was obtained from all participants or their proxies.

In the present study, we used data from the wave (2011–2012) of the cohort. In the wave, a total of 2439 participants who have underwent blood biochemical analysis were selected for our study. For the purpose of this study, 1630 elderly participants met the inclusion criteria in our study after excluded incorrect and missing data, as shown in Additional file 1: Fig. S1.

### Measurement and calculation of BP

All included participants rested for at least 5 min before well-trained researchers took BP measurements for two times on the right arm by using mercury sphygmomanometer (upper arm type; Yuyue, Jiangsu, China). The systolic BP values were performed by Korotkoff phase I and diastolic BP values were performed by phase V. For bedbound participants, the measurements of BP were collected in a recumbent position. The mean value of BP was calculated by the two measurements for further analyses. For our research purposes, diastolic BP ≥ 90 mmHg was defined as diastolic hypertension and systolic BP ≥ 140 mmHg was defined as systolic hypertension.

### Assessment of covariates

Sociodemographic characteristics included age and gender in our study. The other health characteristics covered current smoker, current drinker, activities of daily living, body mass index (BMI), waist circumference, and self-reported diseases diagnosed by a doctor, such as hypertension, heart disease, stroke, diabetes mellitus, and other cardiovascular disease (CVD). Current smoker and current drinker were evaluated by self-report “Do you currently smoke?” and “Do you currently drink alcohol?” Current smoker was categorized as “current smoker” and “not current smoker.” Current drinker was categorized as “current drinker” and “not current drinker”. Activity of daily living was classified as “regular exercise” and “not regular exercise.” Self-reported diseases were classified as “yes” and “not.”

Additionally, for our research purposes, if a participant has a waist circumference at least 80 cm for female and at least 85 cm for male, she or he was defined as obesity. BMI was calculated as bodyweight (kg) divided by squared body height (m^2^). BMI ≥ 25 kg/cm^2^ was defined as obesity.

### Statistical analysis

The continuous variables in our study that were not normally distributed were analyzed by the Mann–Whitney *U* test and then are expressed as the median (interquartile range [IQR]). The Chi-square test were used to analyze categorical variables and were expressed as *n* (%).

First, we implemented a smooth curve to estimate blood MDA levels and systolic and diastolic BP screening in 2011–2012 wave. The smooth curve could fully demonstrate the association of blood MDA levels with BP. Then, stratification analyses were performed to evaluate association between blood MDA levels and systolic and diastolic hypertension risk by adding “obesity (BMI ≥ 25 kg/cm^2^)” and “obesity (waist circumference, male ≥ 90 cm or female ≥ 85 cm)” as a covariate respectively. In these stratification analyses, conditional logistic regression analysis was also used to analyze associations between blood MDA with systolic and diastolic hypertension risk, respectively. The modification effects of “BMI” and “waist circumference” on the relationships between blood MDA levels and diastolic and systolic hypertension were evaluated. All of the analyses were performed by using EmpowerStats 3.0. *P* value less than 0.05 was considered to be significant statistically.

## Results

### Characteristics of participants

As shown in Additional file 2: (Table S1), the median age of participants was 84 years. 839 of them (51.4%) were male. Median of both systolic and diastolic BP were 140.0 mmHg and 80.0 mmHg, respectively. Blood median MDA levels of these included participants were 4.89 mmol/mL. All participants were divided into two groups according to obesity (BMI ≥ 25 kg/cm^2^). Compared with non-obese participants (BMI < 25 kg/cm^2^), obese participants had significantly higher blood MDA levels, diastolic BP and more exercise, and tended to be younger and female, and were more like to suffer from hypertension, heart disease, and diabetes. Information on blood and urine biomarkers is also described in detail in Additional file 2: (Table S1).

### Elevated blood MDA levels were associated with higher diastolic BP by univariate analysis of smooth curve in non-obese population

**Fig. 1:**
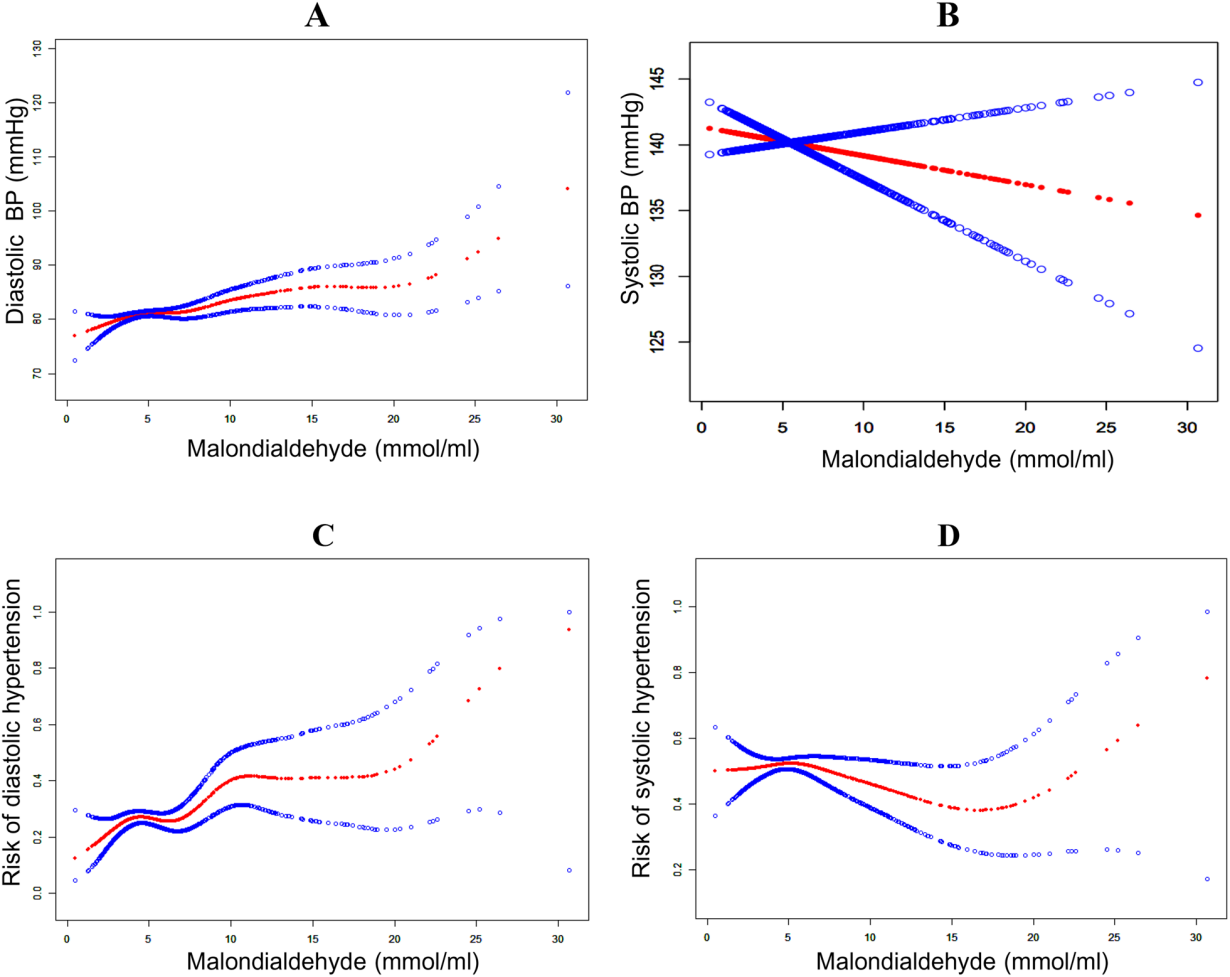
Smooth curve on associations between blood MDA level and BP and risk of hypertension, respectively (Hypertension: diastolic BP ≥ 90 mmHg or systolic BP ≥ 140 mmHg; *MDA* malondialdehyde; *BP* blood pressure)

**Fig. 2:**
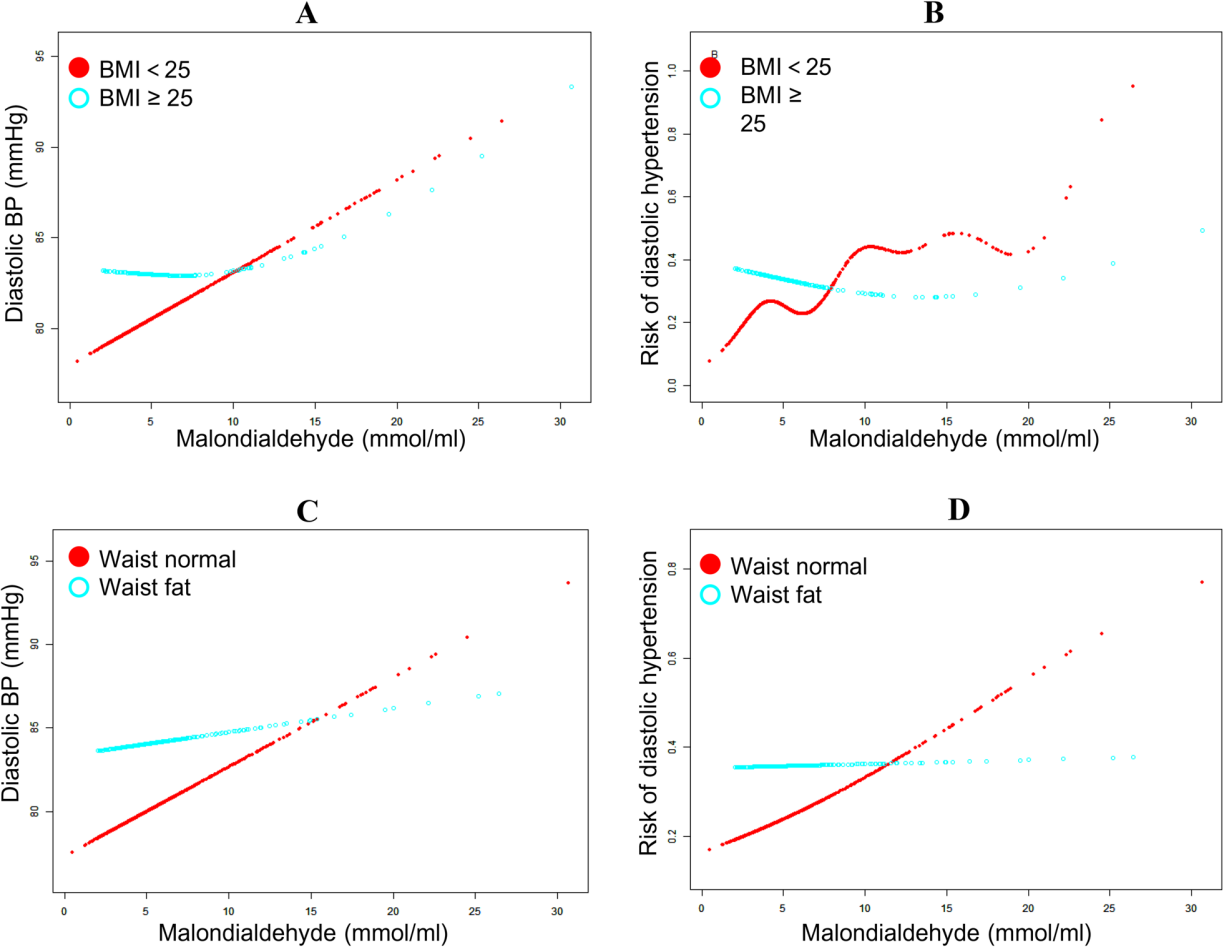
Stratified analysis on associations between blood MDA level and BP and risk of hypertension, respectively (Diastolic hypertension: diastolic BP ≥ 90 mmHg; *MDA* malondialdehyde; *BP* blood pressure; *BMI* body mass index)

We identified the associations between blood MDA levels and systolic and diastolic BP before adjustment of any confounding factors. The results of smooth curve revealed a gradual upward trend on association between blood MDA levels and diastolic BP (Fig. 1A, *P* < 0.001), but not with systolic BP (Fig. 1B, *P* > 0.05). Similar results were analyzed in our study that elevated blood MDA levels were associated with increased risk of diastolic hypertension (diastolic BP ≥ 90 mmHg; Fig. 1C, *P* < 0.001) but not with systolic hypertension (systolic BP ≥ 140 mmHg; Fig. 1D, *P* > 0.05). Furthermore, stratified analysis only demonstrated a significantly gradual upward trend on association between blood MDA levels and diastolic BP and the risk of diastolic hypertension in non-obese participants, rather than in obese participants (Fig. 2A–D). However, the stratified analysis still showed an unrelated association between MDA levels and systolic BP and the risk of systolic hypertension in both of non-obese and obese participants (data not shown).

### Elevated blood MDA levels were associated with diastolic hypertension in non-obese population by multivariate analysis

To eliminate the interference of confounding factors, adjusted analyses were performed. After adjustment for age, gender, current smoker, current drinker, exercise, heart rate, BMI, suffering from diseases, blood biomarkers and urine biomarkers, multivariate linear analysis suggested a significantly positive association MDA levels with diastolic BP (*B* = 0.487, 95% CI 0.276–0.697, *P* < 0.001) but not with systolic BP (*B* = − 0.219, 95% CI − 0.620–0.182, *P* = 0.284) in Additional file 2: (Table S2). Conditional logistic regression analysis suggested elevated MDA levels were still associated with increased risk of diastolic hypertension (*OR* = 1.079, 95% CI 1.039–1.122, *P* < 0.001, Model 3) rather than with systolic hypertension (*OR* = 0.978, 95% CI 0.943–1.015, *P* = 0.247, Model 3) in Table 1 after adjusting all confounding factors. These results are consistent with smoothing curve analysis.

**Table 1:** Multivariate logistic regression analysis for relationship between blood MDA level and risk of hypertension

Variables	Crude	Model 1	Model 2	Model 3
Diastolic hypertension	1.082 (1.043–1.121)	1.083 (1.045–1.124)	1.091(1.051–1.132)	1.079 (1.039–1.122)
*P* Value	< 0.001	< 0.001	< 0.001	< 0.001
Systolic hypertension	0.978 (0.944–1.013)	0.978(0.944–1.012)	0.977 (0.943–1.012)	0.978 (0.943–1.015)
*P* Value	0.207	0.206	0.194	0.247

Crude: Adjusted for age and gender. Model 1: Adjusted for age, gender, current smoker, current drinker, and exercise. Model 2: Adjusted for age, gender, current smoker, current drinker, exercise, heart rate, BMI, and suffering from diseases. Model 3: Adjusted for age, gender, current smoker, current drinker, exercise, heart rate, BMI, suffering from diseases, blood biomarkers and urine biomarkers. Hypertension: diastolic BP ≥ 90 mmHg or systolic BP ≥ 140 mmHg

*MDA* malondialdehyde; *BP* blood pressure; *BMI* body mass index

Furthermore, association between blood MDA levels and hypertension risk was performed by stratified analysis using “BMI” and “waist circumference” as covariate, respectively. We found that elevated blood MDA levels contributed to raised risk of diastolic hypertension in participants with BMI < 25 or waist normal (male < 90 cm or female < 85 cm), rather than in participants with BMI ≥ 25 or waist fat (male ≥ 90 cm or female ≥ 85 cm). Obesity had significant modification effects on association between MDA levels and diastolic hypertension risk for BMI (interaction *P* = 0.015) in Table 2 and for waist circumference (interaction *P* = 0.016) in Table 3. However, obesity had no modification effect on association between MDA levels and systolic hypertension risk for BMI (interaction *P* = 0.790) and for waist circumference (interaction *P* = 0.660).

**Table 2:** Multivariate logistic regression analysis for relationship between blood MDA level and risk of hypertension stratified by “BMI”

Subgroup	Diastolic hypertension			Systolic hypertension		
	OR, 95%CI	*P* Value	*P* for Interaction	OR, 95%CI	*P* Value	*P* for Interaction
BMI < 25 (kg/cm^2^)	1.105 (1.058–1.156)	< 0.001	0.015	0.969 (0.929–1.011)	0.151	0.790
BMI ≥ 25 (kg/cm^2^)	0.968 (0.886–1.058)	0.472		0.971 (0.893–1.055)	0.488	

Adjusted for age, gender, current smoker, current drinker, exercise, heart rate, BMI, suffering from diseases, blood biomarkers and urine biomarkers. Hypertension: diastolic BP ≥ 90 mmHg or systolic BP ≥ 140 mmHg; Obesity: BMI ≥ 25

*MDA* malondialdehyde; *BP* blood pressure; *BMI* body mass index

**Table 3:** Multivariate logistic regression analysis for relationship between blood MDA level and risk of hypertension stratified by “waist circumference”

Subgroup	Diastolic hypertension			Systolic hypertension		
	OR, 95%CI	*P* Value	*P* for Interaction	OR, 95%CI	*P* Value	*P* for Interaction
Waist normal (Male < 90 cm or Female < 85 cm)	0.543 (0.298–0.789)	< 0.001	0.016	− 0.313 (− 0.779–0.153)	0.188	0.660
Waist fat (Male ≥ 90 cm or Female ≥ 85 cm)	0.078 (− 0.324–0.479)	0.704		− 0.156 (− 0.931–0.619)	0.693	

Adjusted for age, gender, current smoker, current drinker, exercise, heart rate, BMI, suffering from diseases, blood biomarkers and urine biomarkers. Hypertension: DBP ≥ 90 mmHg or SBP ≥ 140 mmHg; Obesity: waist circumference in male ≥ 90 cm or female ≥ 85 cm

*MDA* malondialdehyde; *BMI* body mass index

## Discussion

In this cross-sectional study, after adjustment for important identified confounders, smooth curves presented a curve-rising association of blood MDA levels with diastolic BP among adults aged 65 years and older in China. Specifically, higher values of blood MDA predicted an elevated risk of diastolic hypertension in the elderly population. After we explored this association on obesity-stratified analysis of participants, the results supported a modification effect of obesity on the association that elevated blood MDA levels were independently associated with increased risk of diastolic hypertension in non-obese participants but not in obese participants.

In recent years, studies have found that the causes of hypertension are complex; in addition to age, hyperlipidemia, diabetes, obesity, smoking and drinking, staying up late, and others, ROS are the main culprit [20]. MDA, a main end product of lipid peroxidation by ROS, is commonly used as a marker of oxidative status and elevated blood levels of MDA were related to the excessive formation of ROS [21]. It can deactivate NO and also inhibit NO production in the vascular endothelium, which plays a vital role on vasodilation [22]. The imbalance of vasoconstriction and relaxation is the vital pathological mechanism of essential hypertension [1]. Indeed, elevated blood concentrations of MDA in hypertensive patients have been reported previously [1, 13, 14]. In hypertensive animal model or human body, the increase of oxidative stress and subsequent excessive production of ROS promote the elevated concentration of blood MDA. One previous study has demonstrated that patients with essential hypertension have lower NO levels and elevated MDA levels caused by excessive oxidative stress, compared with normotensive individuals [23]. They drew a conclusion that there was a negative correlation between serum NO levels and arterial pressure and a positive correlation between serum MDA levels and mean arterial pressure [23]. Another study also suggested that patients with essential hypertension had elevated concentrations of MDA and indicated increased lipid peroxidation during physical exercise [24]. Considering the negative effect of oxidative stress on vascular endothelial dysfunction which is closely related to the increase diastolic BP caused by impaired vasodilation, we analyzed the relationship between blood MDA levels and risk of systolic and diastolic hypertension, respectively. As we expected, our results showed that the blood MDA levels significant and positively associated with diastolic BP, which is consistent with previous conclusions[23–25] Differently, we did not observed a significant association between blood MDA levels and systolic BP. The insignificant association between serum MDA and systolic BP, however, seems to be consistent with the pathological mechanism that oxidative stress had no significant effect on systolic BP level that was mainly affected by cardiac contractility.

Elevated ROS levels were intricately linked to obesity and its associated pathologies, such as type 2 diabetes, insulin resistance, hyperinsulinemia, increased mobilization of fatty acids, or increased concentration of catecholamines [6–11]. The excess supply of energy substrates in obesity was believed to cause mitochondrial dysfunction and activate ROS signaling, which may underlie insulin resistance and diabetes [24]. Some basic studies suggested that overexpression of mitochondrial catalase and mitochondrially targeted antioxidants can preserve insulin sensitivity in rats with high-fat diets [26]. Both NF-қB and c-Jun N-terminal kinases (JNKs) have been linked to obesity and obesity-induced insulin resistance and are notably regulated by ROS. In the presence of high ROS levels, activated JNKs partly underlie insulin resistance in individuals with obesity [27]. Activated NF-қB highly regulated by ROS has also been shown to alter insulin sensitivity [28]. Furthermore, many existing clinical studies have demonstrated that blood concentrations of ROS were increased in diabetes mellitus and even found in the atherosclerotic plaque deposits promoted by diabetes [29]. Here, stratification analyses were used to evaluate interactions of obesity on the independent association between blood MDA levels and hypertension risk by, respectively, adding “BMI” and “waist circumference” as covariates. Importantly, our results showed that elevated MDA levels were associated with increased risk of diastolic hypertension in non-obese participants rather than in obese participants after adjusting related confounding factors. We found interactions on the association between MDA levels and diastolic hypertension risk for BMI (interaction *P* = 0.015) and for waist circumference (interaction *P* = 0.016) were significant. This phenomenon is very well explained by that the higher level of oxidative stress in obese individuals made it not significant on correlation between blood MDA and diastolic hypertension. Consistently, serum MDA levels were still not associated with risks of systolic hypertension in participants with obesity and normal weight, respectively, and interactions for BMI (interaction *P* = 0.790) and for waist circumference (interaction *P* = 0.660) were not significant.

Our study have some obvious strengths. Firstly, our study results were from an elderly population of CLHLS, which is a prospective, longitudinal, community-based study. This was first national cohort with a Chinese old population and has the high-quality data, including demographic and socioeconomic characteristics, social and behavioral risk factors, and enough biochemical indexes, with a multistage cluster sampling approach. Secondly, in our cross-sectional study, we firstly found an independent association between blood MDA levels presented a curve-rising association with risk of diastolic hypertension but not with systolic BP among adults aged 65 years and older. Specifically, the independent association only exists in non-obese individuals. Thirdly, enough confounding factors, including demographic characteristics, social and behavioral risk factors, and enough biochemical indexes, were adjusted by correction analysis.

Of course, our study also has several limitations. First, all blood tests in our results were only measured once due to cross-sectional analysis. Information regarding the long-term change of MDA level was not available, which may lead to level of MDA inaccurate and may further make results of our analysis biased. Moreover, although more than 9000 individuals were included into CLHLS study, only more than 2439 individuals participated in blood biochemical tests and final 1630 individuals were included in our study because about 800 individuals were excluded due to the absence of important variables. We did not further analyze these excluded individuals.

In conclusion, our results indicated that raised blood levels MDA were associated with elevated risk of diastolic hypertension in non-obese population, which is consistent with previous mechanism studies that oxidative damage contributed to pathological progress of diastolic hypertension. Blood MDA might be an early predictor of diastolic hypertension risk in the future. However, future longitudinal studies need to further confirm our results.

## Supplementary Information

**Additional file 1: Figure S1**. Flow chart of included study population.

**Additional file 2: Table S1**. Characteristics of participants (n=1630). **Table S2**. Multivariate linear regression analysis for relationship between blood MDA level and BP, respectively.

## Data Availability

Study data were from the CLHLS study, which is a prospective, longitudinal, community-based study. ^20^ The datasets used and/or analyzed during the current study are available from the corresponding author on reasonable request.
