# Supplementary material for: Obesity difference on association blood malondialdehyde level and diastolic hypertension in the elderly population: a cross-sectional analysis
Source: Eur J Med Res. 2023 Jan 24;28:44. doi: 10.1186/s40001-022-00983-7 (PMC9872357; doi:10.1186/s40001-022-00983-7)
Supplement: Supplementary file 1 — Additional file 1: Figure S1. Flow chart of included study population. [file 40001_2022_983_MOESM1_ESM.pptx]

## Slide 1
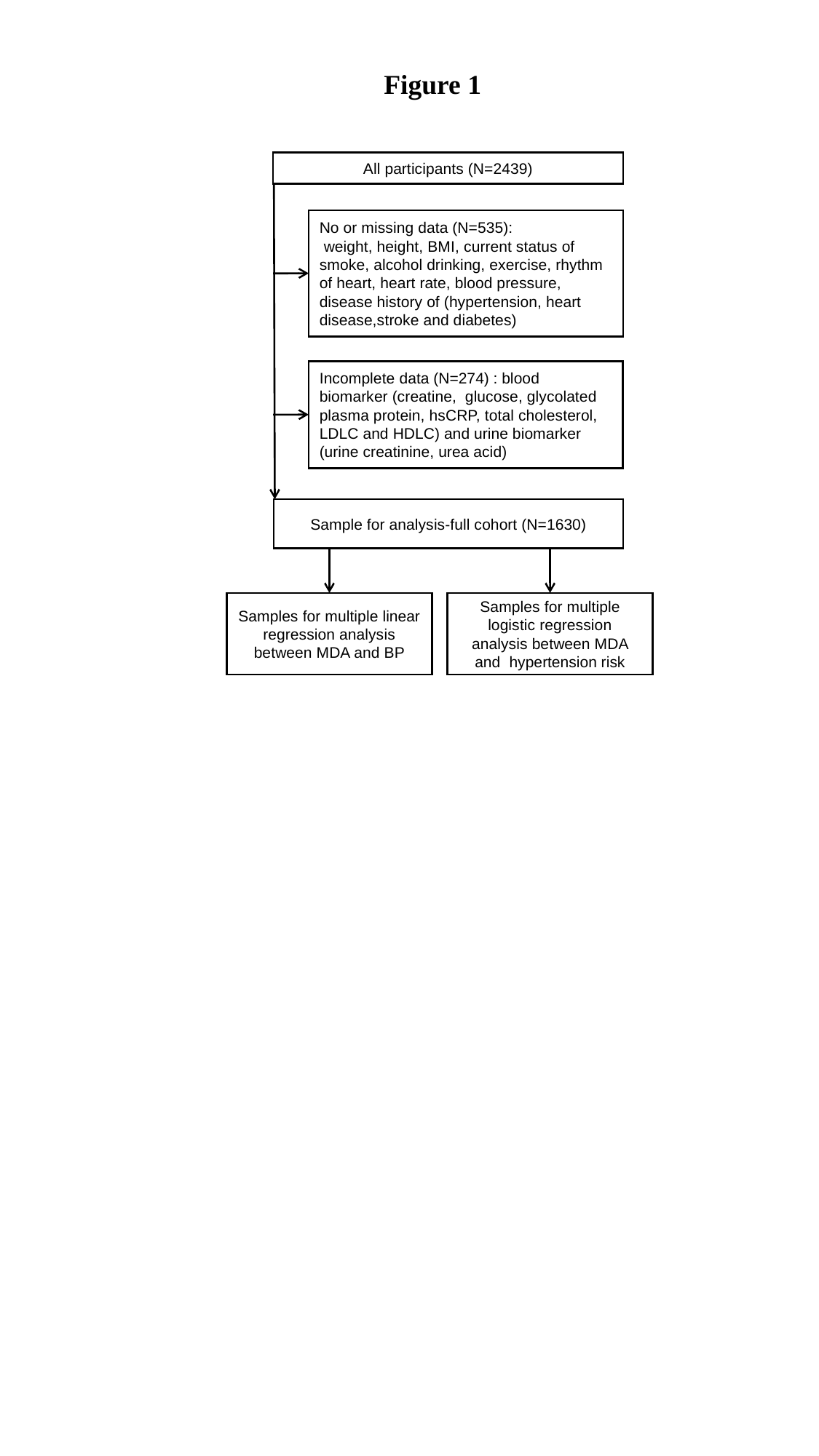

Figure 1
All participants (N=2439)
No or missing data (N=535):
 weight, height, BMI, current status of smoke, alcohol drinking, exercise, rhythm of heart, heart rate, blood pressure, disease history of (hypertension, heart disease,stroke and diabetes)
Incomplete data (N=274) : blood biomarker (creatine, glucose, glycolated plasma protein, hsCRP, total cholesterol, LDLC and HDLC) and urine biomarker (urine creatinine, urea acid)
Sample for analysis-full cohort (N=1630)
Samples for multiple linear regression analysis between MDA and BP
Samples for multiple logistic regression analysis between MDA and hypertension risk
