# Supplementary material for: Obesity difference on association blood malondialdehyde level and diastolic hypertension in the elderly population: a cross-sectional analysis
Source: Eur J Med Res. 2023 Jan 24;28:44. doi: 10.1186/s40001-022-00983-7 (PMC9872357; doi:10.1186/s40001-022-00983-7)
Supplement: Supplementary file 2 — Additional file 2: Table S1. Characteristics of participants (n=1630). Table S2. Multivariate linear regression analysis for relationship between blood MDA level and BP, respectively. [file 40001_2022_983_MOESM2_ESM.doc]

| **Supplementary materials Table 1**. Characteristics of participants (n=1630) | | | | | |
| --- | --- | --- | --- | --- | --- |
| **Variables** | **All participants**  **(n=1630)** | **BMI＜25**  **(n=1381)** | **BMI≥25**  **(n=149)** | **P value** | |
| Age (years) | 84 (73-95) | 85 (75-96) | 69 (77-85) | ＜0.001 | |
| Gender (male), n (%) | 839 (51.4) | 727 (52.6) | 112 (44.9) | ＜0.001 | |
| Waist circumference (cm) | 80 (73-87) | 78 (72-84) | 94 (88-100) | ＜0.001 | |
| BMI (kg/cm^2^) | 20.93 (18.59-23.78) | 20.28 (18.14-22.44) | 27.32 (26-29.24) | ＜0.001 | |
| Average systolic BP (mmHg) | 140.0 (122.5-156.13) | 140 (122-155) | 140 (130-159.5) | 0.045 | |
| Average diastolic BP (mmHg) | 80.0 (72.5-90.0) | 80.0 (72.0-90.0) | 82.5 (75.0-91.0) | 0.003 | |
| Current smoker, n (%) | 291 (17.8) | 247 (17.8) | 44 (17.6) | 0.009 | |
| Current drinker, n (%) | 295 (18.0) | 240 (17.3) | 55 (22.0) | 0.15 | |
| Regular exercise, n (%) | 285 (17.4) | 225 (16.2) | 60 (24.0) | 0.001 | |
| Heart rate (min) | 74(67-81) | 74(67-81) | 72(65.5-80) | 0.143 | |
| **Suffering from diseases** |  |  |  |  | |
| Hypertension, n (%) | 445 (27.3) | 349 (25.2) | 96 (38.5) | ＜0.001 | |
| Heart disease, n (%) | 127 (7.7) | 92 (6.6) | 35 (14.0) | ＜0.001 | |
| Stroke, n (%) | 122 (7.4) | 97 (7.0) | 25 (10.0) | 0.39 | |
| Diabetes, n (%) | 42 (2.5) | 27 (1.9) | 15 (6.0) | 0.02 | |
| Rhythm of heart (irregular), n (%) | 155(9.5) | 126(9.1) | 29(11.6) | 0.11 | |
| **Blood biomarkers** |  |  |  |  | |
| HsCRP (mg/L) | 0.9 (0.40-2.3) | 0.8 (0.4-2.3) | 1.0 (0.5-2.4) | 0.217 | |
| Urea nitrogen (mmol/L) | 6.6 (5.5-7.9) | 6.6 (5.5-7.89) | 6.7 (5.5-8.0) | 0.41 | |
| Creatine (mmol/L) | 77.0 (64.0-91.0) | 77.0 (64.0-92.0) | 76.0 (64.5-88.5) | 0.27 | |
| Total cholesterol (mmol/L) | 4.3 (3.6-4.9) | 4.2 (3.6-4.9) | 4.6 (3.8-5.2) | ＜0.001 | |
| HDL-C (mmol/L) | 1.3 (1.1-1.5) | 1.3 (1.1-1.5) | 1.13 (1.0-1.4) | ＜0.001 | |
| LDL-C (mmol/L) | 2.5 (2.0-3.0) | 2.5 (2.0 -3.0) | 2.8 (2.2-3.3) | ＜0.001 | |
| Glucose (mmol/L) | 4.5 (3.7-5.2) | 4.4 (3.7-5.1) | 4.7 (4.2-5.5) | ＜0.001 | |
| Glycolated protein (mmol/L) | 235.8 (218.7-254.7) | 234.9 (217.8-253.8) | 240.3 (221.9-263.3) | 0.005 | |
| Urea acid (umol/L) | 277.7 (228.1-338.9) | 274.7 (225-336.45) | 292.4 (245.7-355.6) | ＜0.001 | |
| MDA (mmol/mL) | 4.89 (3.94-5.90) | 4.82 (3.88-5.84) | 5.32 (4.39-6.29) | ＜0.001 | |
| **Urine biomarkers** |  |  |  |  | |
| Urine microalbumin (mg/l) | 4.4 (0.8-16.6) | 4.4 (0.84-16.1) | 4.1 (0.8-18.9) | 0.622 | |
| Urine creatinine (umol/L) | 93.1 (57.6-140.9) | 90.6 (53.3-137.8) | 108.0 (71.7-153.4) | ＜0.001 | |
| BMI: body mass index; BP: blood pressure; MDA: malondialdehyde; hsCRP: high sensitivity C-reactive protein; LDL-C: low-density lipoprotein cholesterol; HDL-C: high-density lipoprotein cholesterol; MDA: malondialdehyde. | | | | |  |

| **Supplementary materials Table 2**. Multivariate linear regression analysis for relationship between blood MDA level and BP respectively. | | | | | | | | |
| --- | --- | --- | --- | --- | --- | --- | --- | --- |
| **Variables** | **Diastolic BP** | | | | **Systolic BP** | | | |
|  | **B** | **Sβ** | **B 95% CI** | ***P* Value** | **B** | **Sβ** | **B 95% CI** | ***P* Value** |
| Not adjusted | 0.534 | 0.127 | 0.331-0.737 | ＜0.001 | -0.294 | 0.036 | -0.688-0.099 | 0.143 |
| Adjusted for age and gender | 0.522 | 0.124 | 0.318-0.725 | ＜0.001 | -0.274 | -0.034 | -0.663-0.115 | 0.167 |
| Plus current smoker, current drinker and exercise | 0.521 | 0.124 | 0.317-0.725 | ＜0.001 | -0.266 | -0.033 | -0.655-0.123 | 0.180 |
| Plus history of diabetes | 0.521 | 0.124 | 0.316-0.725 | ＜0.001 | -0.267 | -0.033 | -0.656-0.122 | 0.178 |
| Plus history of stroke | 0.521 | 0.124 | 0.316-0.726 | ＜0.001 | -0.278 | -0.034 | -0.668-0.111 | 0.162 |
| Plus history of heart disease | 0.524 | 0.124 | 0.319-0.728 | ＜0.001 | -0.279 | -0.034 | -0.669-0.110 | 0.160 |
| Plus rhythm of heart (irregular) | 0.539 | 0.128 | 0.335-0.744 | ＜0.001 | -0.274 | -0.034 | -0.665-0.116 | 0.168 |
| Plus heart rate | 0.557 | 0.132 | 0.351-0.762 | ＜0.001 | -0.294 | -0.036 | -0.687-0.099 | 0.142 |
| Plus BMI | 0.554 | 0.131 | 0.348-0.760 | ＜0.001 | -0.295 | -0.036 | -0.688-0.098 | 0.141 |
| Plus blood creatine | 0.525 | 0.125 | 0.318-0.733 | ＜0.001 | -0.242 | -0.030 | -0.638-0.154 | 0.231 |
| Plus urine creatinine | 0.513 | 0.122 | 0.306-0.720 | ＜0.001 | -0.268 | -0.033 | -0.662-0.127 | 0.184 |
| Plus blood urea acid | 0.508 | 0.120 | 0.300-0.715 | ＜0.001 | -0.207 | -0.026 | -0.603-0.188 | 0.304 |
| Plus blood glucose | 0.509 | 0.121 | 0.301-0.717 | ＜0.001 | -0.207 | -0.026 | -0.603-0.189 | 0.305 |
| Plus blood glycolated protein | 0.513 | 0.122 | 0.305-0.720 | ＜0.001 | -0.203 | -0.025 | -0.598-0.193 | 0.315 |
| Plus blood hsCRP | 0.533 | 0.126 | 0.324-0.742 | ＜0.001 | -0.132 | -0.016 | -0.529-0.265 | 0.515 |
| Plus blood total cholesterol | 0.503 | 0.119 | 0.293-0.713 | ＜0.001 | -0.198 | -0.024 | -0.597-0.201 | 0.331 |
| Plus blood LDL-C | 0.511 | 0.121 | 0.301-0.721 | ＜0.001 | -0.188 | -0.023 | -0.588-0.212 | 0.212 |
| Plus bolld HDL-C | 0.487 | 0.115 | 0.276-0.697 | ＜0.001 | -0.219 | -0.027 | -0.620-0.182 | 0.284 |
| MDA: malondialdehyde; BP: blood pressure; BMI: body mass index; hsCRP: high sensitivity C-reactive protein; LDL-C: low-density lipoprotein cholesterol; HDL-C: high-density lipoprotein cholesterol. | | | | | | | | |
